# The calm during the storm: Snowfall events decrease the movement rates of grey wolves *(Canis lupus)*

**DOI:** 10.1371/journal.pone.0205742

**Published:** 2018-10-31

**Authors:** Amanda Droghini, Stan Boutin

**Affiliations:** Department of Biological Sciences, University of Alberta, Edmonton, AB, Canada; Michigan Technological University, UNITED STATES

## Abstract

Mammalian predators encounter unique hunting challenges during the winter as snow increases the cost of locomotion and influences predator-prey interactions. Winter precipitation may also affect predators’ ability to detect and pursue prey. We investigated the effects of snowfall events on grey wolves (*Canis lupus*) in a boreal forest ecosystem in northeastern Alberta, Canada. We predicted that wolves would respond to snowfall events by reducing their travel speed and the time they spent travelling. Over the course of two winters, we used remote cameras to identify localized snowfall events and estimate snow depth. We used telemetry data from 17 wolves to calculate travel speed and time spent travelling versus resting. Data were categorized by time of day (night versus day) and time since snowfall events, and analyzed using linear and logistic regression mixed-effects models. We found that wolves were less likely to travel on dates of snowfall events than any date prior to or after an event. Wolves also travelled slower during snowfall events, but only when compared to their travel speed 24 hours before. Effects were most pronounced at night, when movements appeared to be consistent with hunting behavior, and activity levels resumed within 24 hours of a snowfall event. Including snow depth as a variable did not improve model fit. Collectively, our findings suggest that wolves’ response is not driven by increased hunting success or by energetic considerations resulting from increased snow depth. Instead, we propose that wolves reduce their activity levels because precipitation dampens hunting success. Snowfall events may impact wolves’ ability to detect prey and changes in prey behavior could also lead to decreased encounter rates. We encourage scientists to further investigate the effects of short-term weather events on movement rates and predator-prey interactions.

## Introduction

For many northern mammals, winter is a time when food is scarce and the costs of thermoregulation are high [1,2]. Snow can interact with these already challenging conditions to affect individual health and behavior, population dynamics, and interactions between predators and prey species [3–6]. For example, snow impedes movement and increases the energetic cost of locomotion [3,7,8], it restricts access to key habitats and forage [9–11], and it makes certain individuals and species more vulnerable to starvation and predation [12,13]. Mammals that are active throughout the winter have evolved a suite of morphological and behavioral strategies to overcome this seasonal challenge [1,2,14]. Individuals reduce their activity levels, change their gait, and use compacted routes such as frozen rivers and snowmobile trails to minimize the energetic costs of travelling in snow [3,15–17]. Some ungulate populations seasonally shift their home range, moving to lower elevations where snow is less deep and forage is more accessible, and predators follow suit [11,18–20].

Most of the research on large mammals and snow has focused on the effects of cumulative snow depth (i.e. as the winter season progresses) or on interannual differences between “mild” and “severe” winters [4,21–26]. Recently, we have begun to appreciate that short-term weather events can also have important implications on animal behavior, energetics, space use, and hunting success [3,9,27,28]. In ungulates, sudden changes in snow conditions, either as a result of snowfall or freeze-thaw events, can engender rapid changes in activity levels and temporary shifts in habitat use [9,27]. To our knowledge, no study has described the effects of snowfall on the behavior of predators, especially those that rely on olfaction to detect prey. Falling snow, like other forms of precipitation, rids the air of scent-producing chemical molecules, making it harder to detect prey [29,30]. Strong or erratic winds associated with snowfall events may also have a negative effect on olfaction by affecting detection distance and search rates [28,31–33]. Lastly, fresh snow acts as a sound insulator and covers up animal tracks [30,34].

Our objective was to assess how snowfall events affect the movements of grey wolves (*Canis lupus*; hereafter wolves) in a boreal forest ecosystem in northeastern Alberta, Canada. As cursorial predators, wolves rely heavily on long-distance travel and on olfaction to hunt [35]. Like many other large mammals, wolves’ movements are impeded by deep snow. Yet several studies have reported that wolves experience greater hunting success in deep snow [12,22,23,36–38]. For ones, wolves are lighter on their feet and more agile in deep snow than many ungulate species, allowing them to overtake prey during the chase [14,39,40]. They also adapt their hunting strategy to target undernourished individuals and vulnerable age classes such as shorter-legged calves [12,13,38,41]. However, it is unknown how snowfall events–rather than on-the-ground snow conditions–affect behavior and hunting success. We hypothesized that snowfall events would have a negative impact on wolves’ sensory perceptions and energy budgets. We therefore predicted that wolves would respond to snowfall events by decreasing their speed and the proportion of time spent travelling during and immediately after a snowfall event. We predicted that the severity of these effects would increase with increasing snow depth and that wolves would be most affected at the time of day when they were hunting the most.

## Materials and methods

### Study area

**Fig 1:**
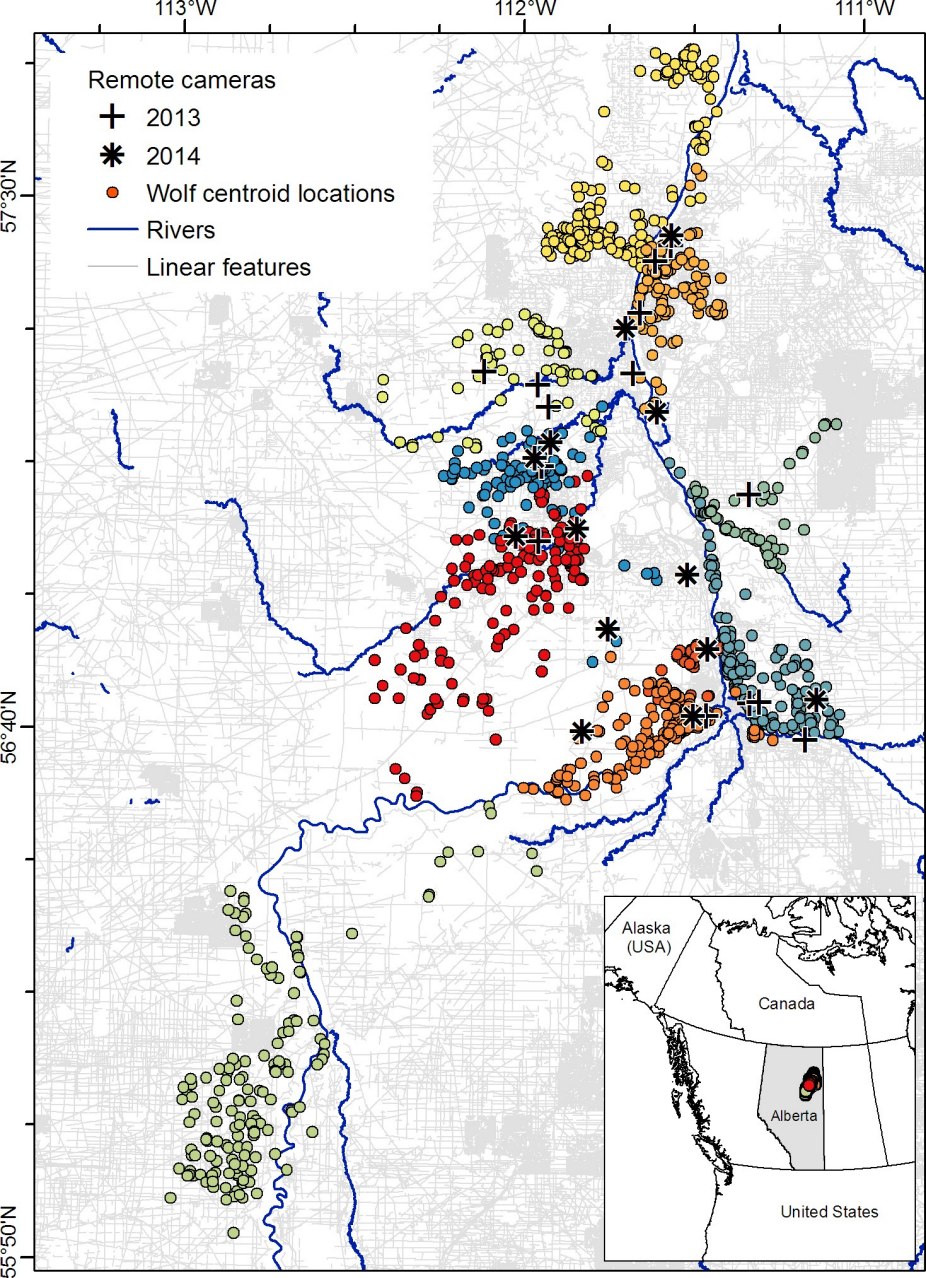
Map of our study area in northeastern Alberta, Canada, near the town of Fort McMurray. From January to March 2013 and 2014, remote cameras were deployed to identify snowfall events, and 17 grey wolves were equipped with GPS telemetry collars. Location fixes were acquired every 10 or 30 minutes and are summarized here as daily centroid locations. Each color represents a wolf pack (*n* = 9, plus one lone wolf). Major rivers are shown in dark blue, while linear features (mostly seismic lines for oil and gas exploration) are in grey. GIS layers are available from the following sources: linear features from the Alberta Biodiversity Monitoring Institute’s Wall-to-Wall Human Footprint Inventory (http://abmi.ca/home/data-analytics/da-top/da-product-overview/GIS-Land-Surface/HF-inventory.html), rivers from Alberta Environment and Parks (https://maps.alberta.ca/genesis/rest/services/Base_Water_Feature/Latest/MapServer), and outlines of Canadian provinces and international boundaries from Natural Earth (https://www.naturalearthdata.com/downloads/10m-cultural-vectors/). Modified and reprinted from A. Droghini and S. Boutin, “Snow conditions influence grey wolf (*Canis lupus*) travel paths: the effect of human-created linear features” Canadian Journal of Zoology 96(1):41. Original copyright 2018. doi: 10.1139/cjz-2017-0041.

Our study took place from January to March 2013 and 2014 in 8,759 square kilometers (km^2^) of central mixed-wood boreal forest in the Athabasca Oil Sands Region (AOSR) of northeastern Alberta, Canada (56.4°N, 111.1°W; Fig 1). Our study area experiences a mean January temperature of -17°C and a mean annual snowfall of 134 centimeters (cm), mostly falling from October to April [based on 1981–2010 climate averages for the Fort McMurray station, 42]. Industry-related linear features are widespread, reaching a mean density of 1.63 km/km^2^ (Fig 1). In the winter, wolves prey mainly on moose (*Alces alces*) [43,44], which are found at low densities (0.04 to 0.15 moose/km^2^) across the landscape [43,45]. White-tailed deer (*Odocoileus virginianus*) are common and increasing in the AOSR, and serve as an alternative prey source [46]. Snowshoe hare (*Lepus americanus*) and rodents are also consumed, but to a far lesser extent [44].

### Inferring movement behavior from telemetry data

We used GPS telemetry data from 17 wolves from January to March 2013 and 2014. Wolves were equipped with Iridium GPS collars (Lotek Wireless Inc., Newmarket, Ontario, Canada). Animal handling was conducted by an experienced crew and followed protocols approved by the University of Alberta’s Animal Care and Use Committee and the Government of Alberta (Research Permit #54143 and #54187, Wildlife Animal Care Committee Class Protocol #009). Although the wolves we analyzed were part of a larger collaring effort [see 43], we restricted our analyses to individuals with fix rates every 30 minutes (min) or less because estimates of fine-scale movements require fast fix rates [47]. Eighteen individuals had fast-fix collars, but one was excluded from our analyses because of collar failure. The remaining 17 individuals belonged to nine different packs, with the exception of one lone wolf (Fig 1). Three individuals had data for both study years, so we had data on 10 individuals for each year of our study. Of the collars we included, nine were programmed to collect one location every 30 min, and eight collected one location every 10 min.

All data manipulation, including data cleaning, processing, and statistical analyses were conducted in R [48] using the following packages: *dplyr*, *data*.*table*, *ggplot2*, *lme4*, *mixtools*, *MuMIn*, *plyr*, *suncalc*, *tidyr* [49–57]. Data from 10-min collars were thinned to 30 min intervals to enable comparisons across individuals. We then followed the approach of Dickie et al. [58] to separate our telemetry data into two exclusive behaviors: “rest” (slow speed) and “travel” (fast speed). For each individual, we calculated the speed between two consecutive, chronologically ordered GPS points (“steps”). We generated a frequency distribution of the log_10_-transformed speed values, pooled across all individuals (S1 Fig). As expected [58], the histogram revealed a bimodal distribution, which suggested that wolf movements could be classified into at least two discrete behaviors. We fit two normal distributions to our data and visually estimated their intersection point (S1 Fig). Steps < 1.65 meters/minute (m/min) were classified as “rest”, while steps ≥ 1.65 m/min were classified as “travel”. [58].

### Estimating snow depth and snow accumulation

We deployed 14 remote cameras (Reconyx PC900, Reconyx Inc., Holmen, WI, USA) in 2013 and 13 cameras in 2014 (Fig 1). The placement of our cameras was constrained to areas that were accessible by cars and snowmobiles; however, we tried to ensure adequate coverage of our study area (Fig 1). Cameras were deployed in open habitat (canopy cover ≤ 30%) at the beginning of the field season and were programmed to take one photo every day at noon. Upon deployment of each camera, field technicians installed a long metal pole in the ground and measured snow depth at the pole’s location using a meter stick. The pole was marked with black tape every 10 cm (S1 Photo), and the camera’s lens was aimed at this pole. After the photos were downloaded to a computer, we used the poles’ 10 cm markers to estimate daily snow accumulation (change in snow depth over a 24 hour period). We obtained estimates of absolute snow depth by adding the initial snow depth measurement to our estimates of snow accumulation.

### Identifying snowfall events

We defined a snowfall event as an accumulation of ≥ 5 cm in 24 hours (h). There is no strict definition of a “snowfall event”; as a point of reference, Environment Canada issues a “snowfall warning” in Alberta when ≥ 10 cm snow accumulates in 12 h or less [59]. We used a more inclusive definition because our cameras only took pictures once every 24 h, and because snowfall events ≥ 10 cm were rare in our study.

We used snow accumulation data from our remote cameras to identify the dates on which individual wolves experienced snowfall events. To do so, we had to match camera data to telemetry data by date and by location. The first step was to reconcile the camera’s temporal resolution with the resolution of our telemetry data. Because our cameras took one photo every day at noon, their ability to identify the date of a snowfall event was offset by 12 h i.e. snowfall events that took place after 12:00 PM could only be detected on the following day. Telemetry data after 12:00 PM were therefore matched to snow data from the next day. To do so, we created a “camera day” variable for our telemetry data that added +1 day to the “real” date for all fixes occurring after 12:00 PM and used this variable to join our snow data with telemetry data. All subsequent methods and mentions of dates are based on this “camera day” definition.

For each individual wolf and for each date, we identified the camera that was nearest to the mean of its easting and northing coordinates (“daily centroid location”; Fig 1), resulting in one snow depth value and one snow accumulation value for each individual-day combination. Using daily centroids prevented multiple cameras (and therefore multiple values) from being assigned to a wolf for a single day (e.g. in instances where two cameras were close together, or where wolves traveled far distances).

In areas of high human activity, wolf packs may be more active at night to minimize interactions with humans [60,61]. We categorized our telemetry data into “day” (times between sunrise and sunset) and “night” (times between sunset and sunrise) using daily sunrise and sunset times [56] and the coordinates at each daily centroid location.

With this dataset, we identified all dates on which an individual wolf experienced a snow accumulation of 5 cm or more (i.e. a snowfall event) and classified our telemetry data into seven “snowfall categories”. The first six categories were date-based, 24 h periods that spanned from two days before a snowfall (“two_before”) to three days after (“three_after”), including the day of the snowfall event (“day_of_snowfall”). This was the maximum length of time we could analyze while avoiding an overlap between consecutive snowfall events. The seventh and final category served as a control. Controls were created for each wolf by randomly selecting telemetry data from three dates that fell outside of this time window. So, control dates happened at least three days before or four days after a snowfall event. Because of the small sample of snowfall event dates relative to our dataset, the number of observations to include in the control category was chosen to balance the number of observations in the other categories. Data not assigned to any snowfall category and without any snow depth values (0.02% of data points) were omitted. All summary statistics and statistical models were obtained using this subset dataset.

### Statistical analyses

We modelled the effects of snowfall on two movement metrics: travel speed and time spent travelling using mixed-effects models. We analyzed “travel speed” using linear regression, while “time spent travelling” was analyzed using logistic regression. “Travel speed” only included the subset of our data classified as “travel” (i.e. speed > 1.65 m/min). Speed was log_10_-transformed prior to analysis to improve the distribution of the residuals. “Time spent travelling” was defined as the proportion of travel steps relative to the total number of steps. In this case, the proportion of travel steps is a suitable proxy for time because we standardized the length of time between two steps to 30 min (sd = 0.31, range: 19.75–40.92 min).

For each movement metric (“travel speed” and “time spent travelling”), we began with the global model: movement metric ~ snowfall_category * time_of_day + snow_depth. and evaluated whether adding random effects improved model fit [62]. We used an information-theoretic approach for model selection. Because it was biologically reasonable to expect that all explanatory variables could be important (either singly or in combination), the set of candidate models included all combinations of explanatory variables (*n* = 10 models). Final models were chosen based on AIC values, log-likelihood values, and evidence ratios [63]. We estimated regression coefficients and confidence intervals using parametric boot-strapping and *n* = 5,000 simulations.

## Results

### Summary of snow conditions

Remote cameras were deployed for 56.8 ± 12.5 days (mean ± sd). The number of cameras assigned to each individual wolf ranged from 2 to 8. One wolf resided in the extreme southwestern part of our study area and had no cameras nearby (mean distance from camera to daily centroids: 90.1 km). Excluding this individual, cameras were 19.2 ± 12.0 km away from wolves’ centroid locations. Snow depth ranged from 13 to 90 cm and was similar in 2013 and 2014 with a mean of 50.7 cm and 49.3 cm, respectively.

We identified 19 unique snowfall events, for a total of 56 records across 17 individuals. Of these 19 events, four took place in 2014. In 2013, wolves (*n* = 10) experienced a mean of 4.3 events (range: 1–6), whereas wolves in 2014 (*n* = 10) experienced a mean of 1.3 events (range: 1–2). Over the course of our two-year study, we recorded only seven instances of daily snow accumulation ≥ 10 cm. The most severe snowfall event recorded on our cameras resulted in a 16 cm accumulation of snow within 24 h. Snowfall events were highly localized: 9 of the 19 (47%) were experienced by only one individual. Only one snowfall event, resulting in snow accumulation from 5 to 16 cm (depending on the camera), was experienced by all individuals in that year.

### Travel speed

Summary statistics from our raw data indicate a mean travel speed of 26.3 ± 23.8 m/min at night, compared to 20.3 ± 19.9 m/min during the day. When averaged across time of day, wolves covered the least distance on snowfall event dates (10.06 ± 8.92 km/day); mean daily distance for other snowfall categories ranged from 11.30 km/day (“two_before”) to 13.14 km/day (“control”).

Both *time_of_day* and *snowfall_category* were important predictors of travel speed (Table 1). The highest ranked model was a linear combination of *snowfall_category* and *time_of_day*. Three other models had a ΔAIC ≤ 4; however, in the case of nested models, more complex models within a few AIC units of the top model should be scrutinized to determine whether the addition of extra parameters is supported [63]. In our case, models which included the interaction term or the *snow_depth* variable had log-likelihood values which were very close to the value of the top model (Table 1), suggesting that the additions of these variables does not actually improve model fit [63]. Consequently, we focus only on the highest ranked model to derive regression coefficients.

**Table 1:** Model selection results describing wolf travel speed as a function of snow depth, time of day (day versus night), and snowfall category (time since snowfall event). Models were fitted with a random effect structure for each individual wolf (*n* = 17). The structure we specified allows for a by-individual random intercept and random slope over *time_of_day*.

Rank	Formula*	K	log(L)	AIC	ΔAIC	*w*_*i*_
1	snowfall_category + time_of_day	12	-3501.96	7027.93	0.00	0.51
2	snowfall_category * time_of_day	18	-3496.88	7029.75	1.82	0.20
3	snowfall_category + time_of_day + snow_depth	13	-3501.95	7029.90	1.98	0.19
4	snowfall_category * time_of_day + snow_depth	19	-3496.85	7031.71	3.78	0.08
5	time_of_day	6	-3511.88	7035.76	7.83	0.01
6	snowfall_category	11	-3507.28	7036.55	8.62	0.01
7	time_of_day + snow_depth	7	-3511.75	7037.50	9.57	0.00
8	snowfall_category + snow_depth	12	-3507.26	7038.52	10.60	0.00
9	Null model	5	-3517.27	7044.54	16.61	0.00
10	snow_depth	6	-3517.15	7046.30	18.37	0.00

* Dependent variable: Travel speed of grey wolves (log_10_-transformed).

**Fig 2:**
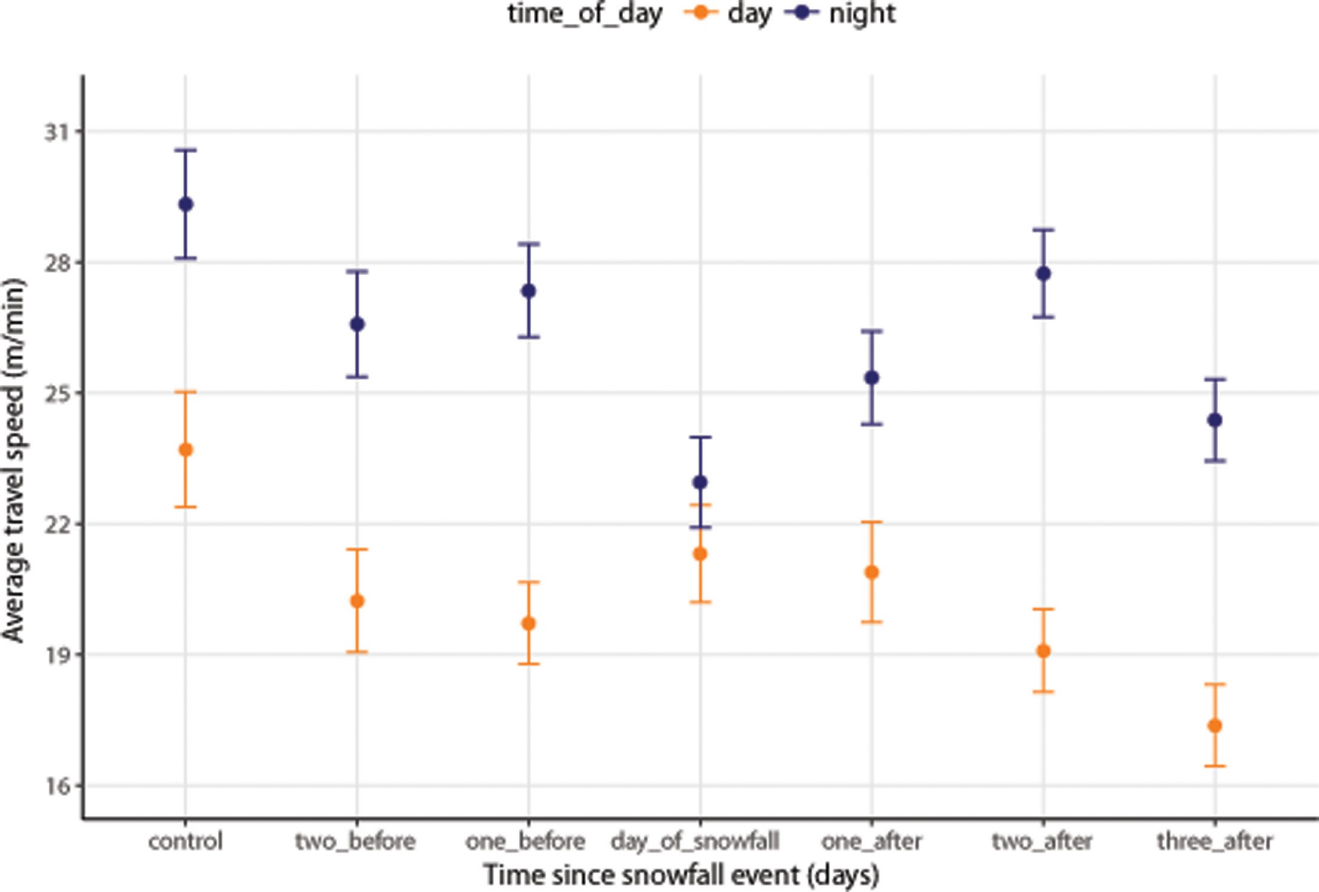
Grey wolves respond to snowfall events by reducing their travel speed, when compared to speeds 24 h before and to random controls. **The effect appears strongest at night.** Data points represent mean values of the raw data across all individuals (*n* = 17). Error bars represent one standard error of the mean.

Regression coefficients indicate that wolves travelled faster at night than during the day (Table 2). Wolves travelled slower on the date of a snowfall event, compared to one day before the event and to control dates, but travel speed during snowfall events was not any different than speeds immediately after, or several days prior to, a snowfall event (Table 2). Although an interaction between time of day and time since snowfall events did not improve model fit (Table 1), the telemetry data suggest that the effect of snowfall events is more pronounced at night than during the day (Fig 2).

**Table 2:** Estimates of regression coefficients, standard error, and 95% confidence intervals for our final model evaluating the effects of snowfall and time of day on travel speed. Because the dependent variable was log_10_-transformed, coefficients were back-transformed using the formula 10exp^*x*^, where *x* is the estimate of interest.

Untransformed coefficients			Transformed coefficients		
Variable^*^	β	Standard error		95% confidence intervals	
			β	Lower limit	Upper limit
Intercept	1.081	0.026	12.049	10.701	13.609
time_of_day: night	0.107	0.028	1.280	1.130	1.459
snowfall_category: control	0.061	0.023	1.152	1.037	1.273
snowfall_category: two_before	0.037	0.023	1.088	0.981	1.211
snowfall_category: one_before	0.045	0.021	1.110	1.006	1.222
snowfall_category: one_after	0.005	0.022	1.012	0.914	1.116
snowfall_category: two_after	0.041	0.022	1.099	0.995	1.210
snowfall_category: three_after	-0.016	0.022	0.964	0.874	1.064

* Dependent variable: Travel speed of grey wolves (log_10_-transformed).

### Proportion of time spent travelling

During the day, the proportion of time spent travelling did not vary much by snowfall categories, ranging from a mean of 0.33 (on control dates) to 0.38 (on snowfall event dates). Nighttime behaviors appear to be more affected by snowfall events. On snowfall event nights, travel comprised 0.27 of behaviors, while mean values for all other snowfall categories were similar to daytime values and ranged from 0.32 to 0.36.

As with our travel speed model, *snowfall_category* and *time_of_day* were important predictors for the amount of time wolves spent travelling. Model selection also supported an interaction between these two terms. Indeed, the only two models that had strong support both included this interaction term (Table 3). The top-ranking model also included the *snow_depth* variable (Table 3); however, including the *snow_depth* variable does not appear to improve model fit because it is within one AIC unit of the second model and has nearly the same log-likelihood value [63]. We therefore selected the second-ranked model as the best approximating model and used it to derive regression coefficients (Table 4).

**Table 3:** Model selection results describing the proportion of travel behavior as a function of snow depth, time of day (day versus night), and snowfall category (time since snowfall event). Models were fitted with a random effect structure, which allowed for a random intercept for each individual wolf (*n* = 17).

Rank	Formula*	K	log(L)	AIC	ΔAIC	*w*_*i*_
1	snowfall_category * time_of_day + snow_depth	16	-10,769.14	21,570.28	0.00	0.62
2	snowfall_category * time_of_day	15	-10,770.63	21,571.25	0.97	0.38
3	snowfall_category + time_of_day + snow_depth	10	-10,787.63	21,595.25	24.97	0.00
4	snowfall_category + time_of_day	9	-10,789.12	21,596.24	25.96	0.00
5	time_of_day + snow_depth	4	-10,799.45	21,606.90	36.62	0.00
6	time_of_day	3	-10,800.63	21,607.26	36.98	0.00
7	snowfall_category + snow_depth	9	-10,796.56	21,611.13	40.85	0.00
8	snowfall_category * time_of_day + snow_depth	8	-10,798.37	21,612.73	42.45	0.00
9	snow_depth	3	-10,808.26	21,622.53	52.25	0.00
10	Null model	2	-10,809.69	21,623.37	53.09	0.00

* Dependent variable: Movement behavior coded as "travel" (1) or "rest" (0).

**Table 4:** Estimates of regression coefficients, standard error, and 95% confidence intervals for our final model evaluating the effects of snowfall and time of day on the proportion of time spent travelling. Coefficients are presented on the logit scale and were back-transformed using the formula exp^*x*^, where *x* is the estimate of interest.

Untransformed coefficients			Transformed coefficients		
Variable*	β	Standard error	β	95% confidence intervals	
				Lower limit	Upper limit
Intercept	-0.467	0.079	0.627	0.544	0.725
time_of_day: night	-0.542	0.086	0.582	0.507	0.664
night × control	0.563	0.125	1.756	1.425	2.187
night × two_before	0.456	0.130	1.578	1.267	1.969
night × one_before	0.365	0.121	1.440	1.172	1.771
night × one_after	0.268	0.123	1.307	1.063	1.608
night × two_after	0.621	0.123	1.862	1.507	2.316
night × three_after	0.546	0.122	1.726	1.410	2.138
snowfall_category: control	-0.243	0.096	0.784	0.658	0.926
snowfall_category: two_before	-0.201	0.100	0.818	0.681	0.975
snowfall_category: one_before	-0.026	0.094	0.974	0.821	1.153
snowfall_category: one_after	-0.025	0.094	0.975	0.82	1.159
snowfall_category: two_after	-0.169	0.096	0.845	0.709	1.006
snowfall_category: three_after	-0.095	0.095	0.909	0.763	1.074

* Dependent variable: Movement behavior coded as "travel" (1) or "rest" (0).

**Fig 3:**
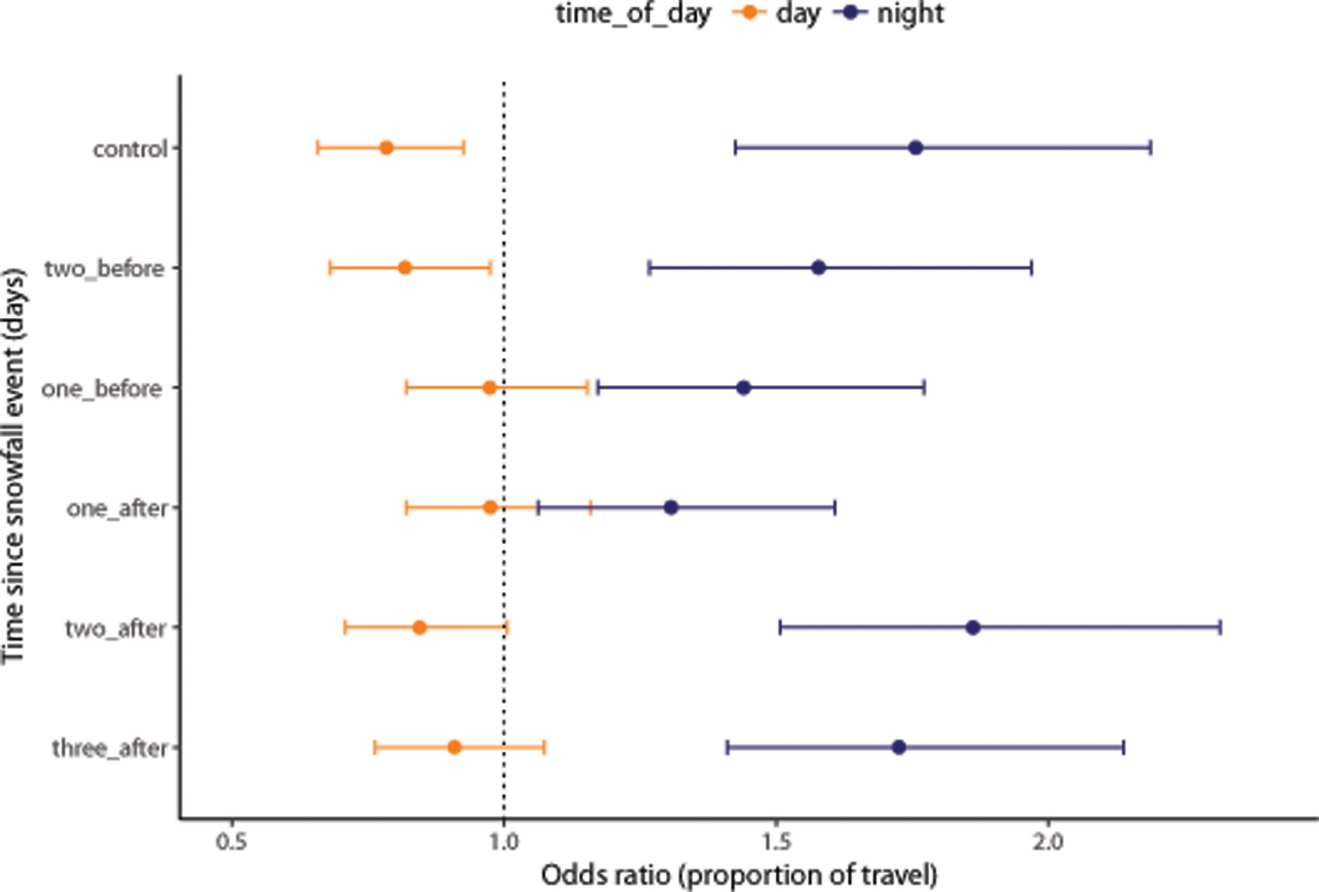
Wolves are least likely to travel on the night of a snowfall event, compared to dates immediately before or after an event. Coefficients were estimated from a logistic regression mixed-effects model. Error bars represent 95% confidence intervals.

Wolves were less likely to be travelling on the night of a snowfall event than they were at any other time (Fig 3). The effect of snowfall events was relatively short-lived. Travel behavior returned to normal the following day and remained likely 48 and 72 h after an event (Fig 3).

## Discussion

On the night of a snowfall event, wolves travelled slower and were less likely to be travelling than on nights before or after a snowfall. Activity levels resumed within 12 h to 24 h. Though short-lived, the effects of snowfall events may be strong enough to impact daily movement rates but this idea has not been explicitly tested. Other studies have documented periods of reduced activity as a result of unfavorable snow conditions [45,64]. In one instance, snow depth was >50 cm and “soft and fluffy” conditions persisted for several weeks; during that period of time, daily distance travelled was nearly half what it was when snow conditions were deep, yet supportive [64]. An interannual study, also located in northeastern Alberta, found that wolves reduced their daily movement rates the year where mean snow values were 20 cm deeper and temperatures were colder [45].

The effects we observed were more pronounced at night than during the day (Figs 2 and 3). Most snowfall events in our study area occur in the evening or overnight, which is also when wolves in our study area appear to be hunting the most: under non-snowfall conditions, they travelled faster at night though the proportion of travel remained roughly the same. There are several reasons why wolves may be travelling slower and travelling less during snowfall events: 1) Wolves are responding to prey behavior. Prey are less active, leading to decreased encounter rates; 2) Falling snow has a negative impact on sensory perceptions, leading to a decreased hunting success; 3) Wolves have higher kill rates during snowfall, leading to more time spent at kill sites and less time travelling; or 4) The energetic cost of movement is too high.

The first two options could explain the patterns we see in our data. They assume that wolves are reducing activity levels during snowfall events because hunting success is low. Although wolves are well-adapted to chasing down prey in deep snow [39,40], encounter rates may be low if wolves have a harder time detecting prey or if prey reduce their movement rates during snowfall events [65]. Wolves in forested areas hunt primarily through olfaction [35]. Snowfall may make it harder to detect prey by ridding the air of scent molecules and also by insulating sound and covering tracks [29,30,34]. Associated weather conditions such as wind and temperature can also influence predators’ ability to detect prey [28,30,33]. We do not know how prey in our study area responded to snowfall events and only a few studies have considered the response of herbivores to short-term weather events elsewhere. Wild boar (*Sus scrofa*) in Sweden and mountain goats (*Oreamnos americanus*) in Alberta’s Rocky Mountains decrease their activity levels in response to snowfall events [9,15]. In alpine chamois (*Rupicapra rupicapra*), snowfall events weakly affected females and did not affect males [66]. Certain events, however, may increase movement rates. Svalbard reindeer (*Rangifer tarandus plathyrynchus*) temporarily increased their activity levels after freezing events made forage inaccessible and reindeer moved to more favorable areas [27]. Exploring how predators and prey respond to the same weather events could provide some interesting insights on predator-prey interactions in northern environments. Understanding how environmental variables affect olfaction would benefit ecological studies while also informing work with scent detection dogs used for conservation purposes.

As an alternative explanation, wolves may travel less because their hunt has borne fruit. Several studies have reported that wolves experience increased hunting success in deep snow [12,36,37,39,41] and wolves may target specific age classes or species depending on snow conditions [12,38,41]. After a successful hunt, wolves stay at kill sites to eat and rest. They typically spend less than 24 h at deer kills [26,43,67] but spend several days at carcasses of larger prey such as moose [41,43,45,67]. Wolves in our study area spent more than 20 h at deer kills and more than two days at moose kills [43]. Wolves in northeastern Alberta also spent a mean of 1.7 days at moose calf kill sites [45]. Thus, regardless of age class or prey species, we would expect low movement rates for one to two days after a snowfall event. Instead, we found no evidence of reduced movement rates the day following a snowfall event (Figs 2 and 3), suggesting that snowfall events do not lead to increased kill rates.

Lastly, if behavior were driven by energetic considerations, we would expect wolves to further reduce their activity as snow depth increases [3,7]. During the two years of study, our cameras recorded a broad range of snow depth values, many of which exceeded the threshold at which wolves are expected to be impeded (40 to 50 cm; [19,64]). Yet snow depth was not a strongly supported predictor variable in either of our models (Tables 1 and 3). Our cameras provided us with localized snow conditions and detected increases in snow depth from one day to the next. Still, they may not reflect the exact snow conditions experienced by wolves. Wolves exhibit strong selection for travel routes on shallow, compact snow, such as frozen rivers, windswept ridges, and snowmobile trails [19,68–70]. A study on coyotes, which exhibit a similar behavior, suggests that microhabitat selection of travel routes can offset the costs of travelling in snow [7]. Travelling as a pack likely confers energetic savings as well. Whether wolves use ploughed or established travel routes more during a snowfall event is unknown, but it has been suggested that wolves increase their use of these features in the winter [61]. Exploring this question would likely require faster fix rates than the 30 min intervals used here [58].

Using remote cameras allowed us to successfully detect highly localized winter weather events. Snowfall events larger than the ones we detected here will likely elicit stronger reductions in activity levels. The type of snow that is falling will also affect energetic costs and predator-prey dynamics. Wet, heavy snow and snow that forms an unsupportive crust dramatically increases the cost of movement and impedes prey’s ability to escape [3,8,71]. Studies where heavy snowfall events are more common, such as alpine and maritime regions or those influenced by lake-effect snow, may be particularly well-suited for testing the hypotheses we outline here.

## Supporting information

S1 Fig Histogram of log_10_-transformed speed values for separating telemetry data into resting and travelling movement behaviors. We followed the approach by Dickie et al. (2017) to isolate travelling behavior in wolves from GPS telemetry data. Our histogram of log_10_-transformed speed values revealed a bimodal distribution, which suggests that wolf movements can be discretized into two behaviors: slow (“rest”) and fast (“travel”). We modelled the density distribution as two Gaussian curves and used the intersection point as a cut-off value. Speeds greater than or equal to 1.65m/min were classified as “travel”, whereas values less than that were classified as “rest”.
(PNG)

S1 Photo Field set-up used to estimate snow depth with remote cameras. We estimated snow depth and snow accumulation by using remote cameras (Reconyx PC900, Reconyx Inc., Holmen, WI, USA) deployed across our study area. Cameras were programmed to take one picture every day at noon and were aimed at poles that were marked with black tape every 10 centimetres. We estimated snow accumulation by counting the number of black lines that were visible from one day to the next. Snow depth was estimated by adding initial snow depth (measured during deployment) to estimates of snow accumulation.
(PDF)
