# Supplementary figures and images for: The calm during the storm: Snowfall events decrease the movement rates of grey wolves (Canis lupus)
Source: PLoS One. 2018 Oct 31;13(10):e0205742. doi: 10.1371/journal.pone.0205742 (PMC6209196; doi:10.1371/journal.pone.0205742)

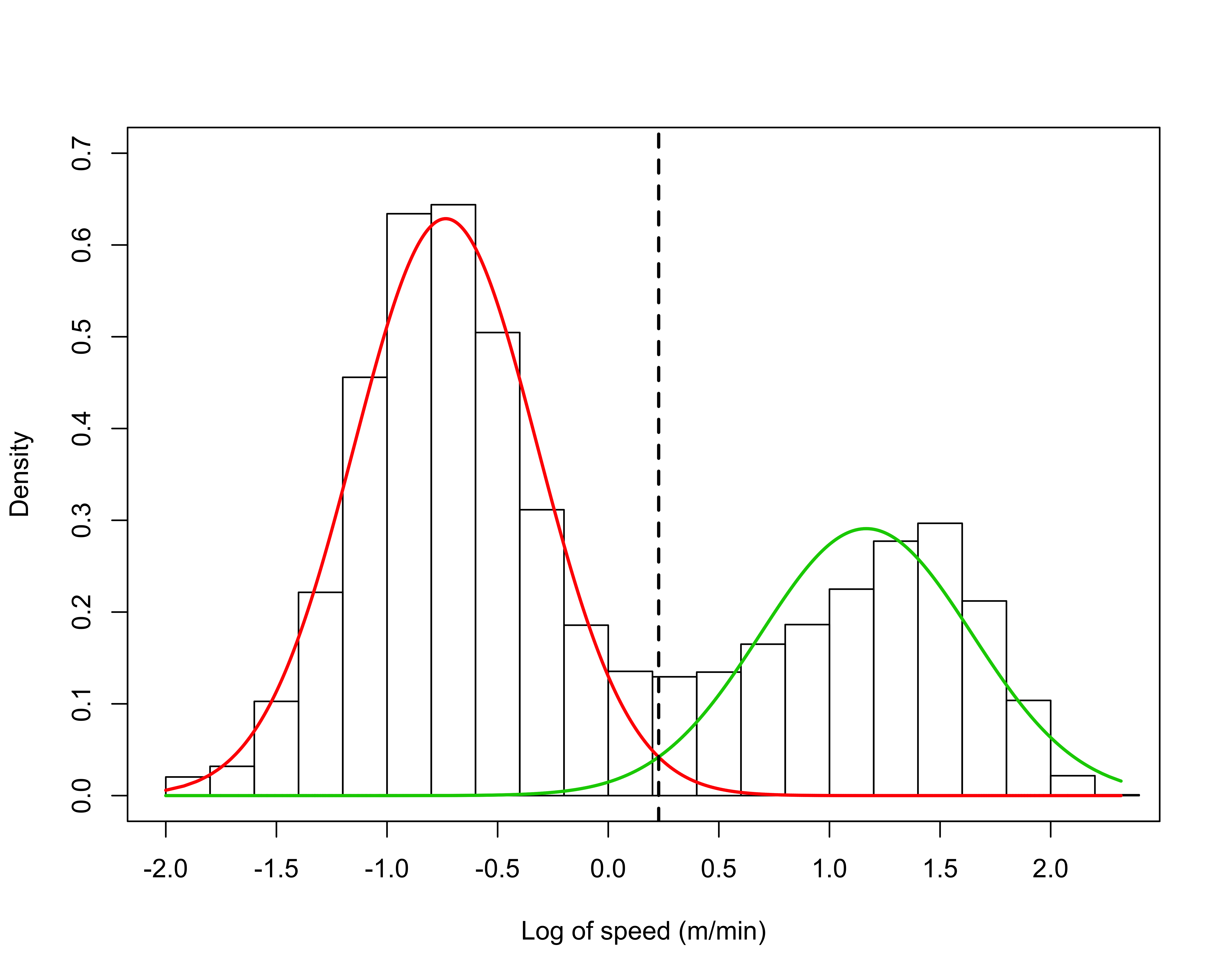

Supplement: S1 Fig — We followed the approach by Dickie et al. (2017) to isolate travelling behavior in wolves from GPS telemetry data. Our histogram of log10-transformed speed values revealed a bimodal distribution, which suggests that wolf movements can be discretized into two behaviors: slow (“rest”) and fast (“travel”). We modelled the density distribution as two Gaussian curves and used the intersection point as a cut-off value. Speeds greater than or equal to 1.65m/min were classified as “travel”, whereas values less than that were classified as “rest”. (PNG) [file pone.0205742.s001.png]
