# Supplementary material for: The calm during the storm: Snowfall events decrease the movement rates of grey wolves (Canis lupus)
Source: PLoS One. 2018 Oct 31;13(10):e0205742. doi: 10.1371/journal.pone.0205742 (PMC6209196; doi:10.1371/journal.pone.0205742)
Supplement: S1 Photo — We estimated snow depth and snow accumulation by using remote cameras (Reconyx PC900, Reconyx Inc., Holmen, WI, USA) deployed across our study area. Cameras were programmed to take one picture every day at noon and were aimed at poles that were marked with black tape every 10 centimetres. We estimated snow accumulation by counting the number of black lines that were visible from one day to the next. Snow depth was estimated by adding initial snow depth (measured during deployment) to estimates of snow accumulation. (PDF) [file pone.0205742.s002.pdf]

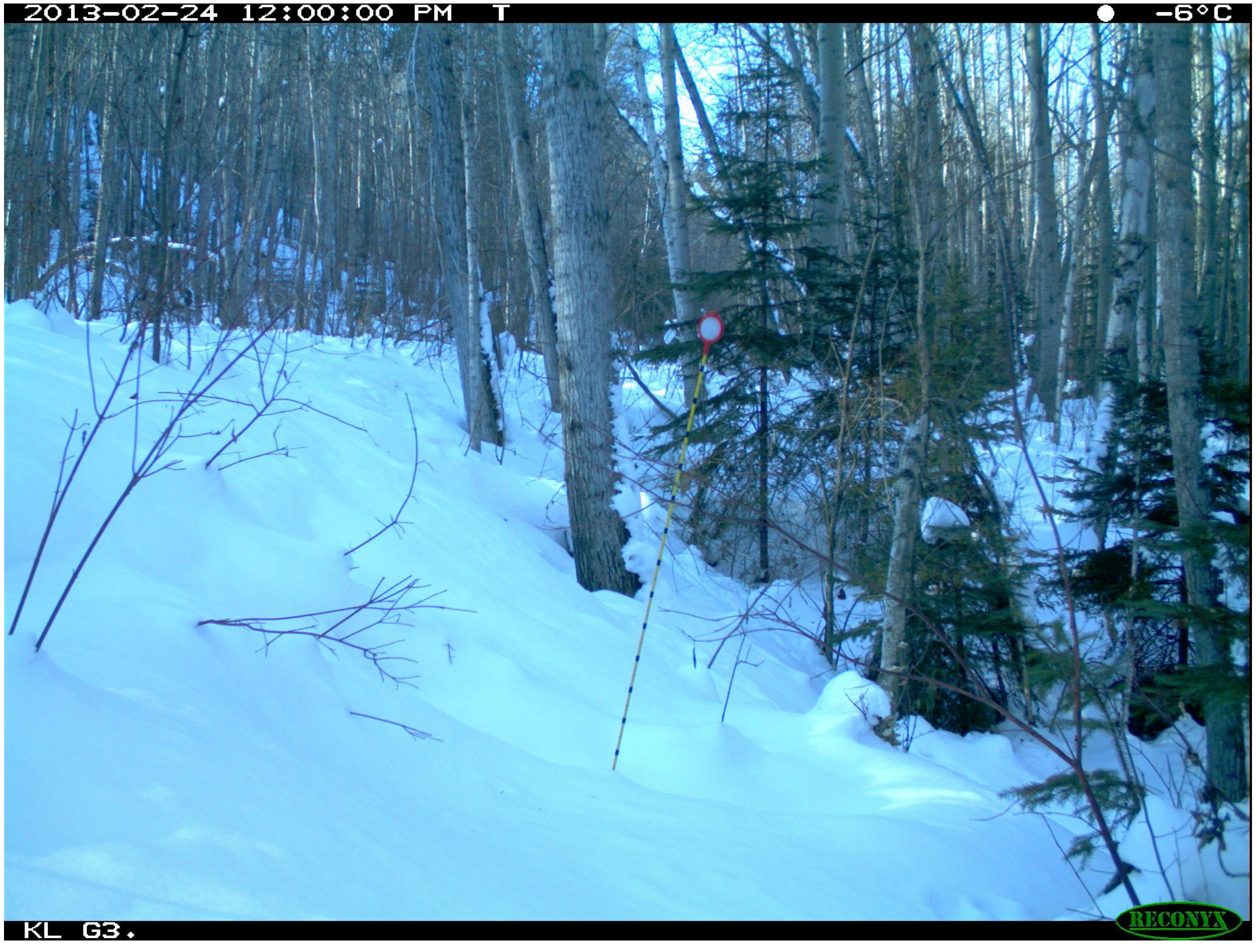

S1 Photo. Field set-up used to estimate snow depth with remote cameras. We estimated snow depth and snow accumulation by using remote cameras (Reconyx PC900, Reconyx Inc., Holmen, WI, USA) deployed across our study area. Cameras were programmed to take one picture every day at noon and were aimed at poles that were marked with black tape every 10 centimetres. We estimated snow accumulation by counting the number of black lines that were visible from one day to the next. Snow depth was estimated by adding initial snow depth (measured during deployment) to estimates of snow accumulation.
